# Trastuzumab in Esophagogastric Cancer: HER2-Testing and Treatment Reality outside Clinical Studies in Germany

**DOI:** 10.1155/2016/1028505

**Published:** 2016-01-28

**Authors:** Kirsten Merx, Manuel Barreto Miranda, Lenka Kellermann, Ulrich Mahlknecht, Oliver Lange, Michael Gonnermann, Ralf-Dieter Hofheinz

**Affiliations:** ^1^TagesTherapieZentrum am Interdisziplinären Tumorzentrum Mannheim und III. Medizinische Klinik, Hämatologie und Onkologie, Universitätsmedizin Mannheim, Universität Heidelberg, 68167 Mannheim, Germany; ^2^Marienhospital Darmstadt, Klinik für Innere Medizin, 64285 Darmstadt, Germany; ^3^Oncology Information Service (OIS), 79098 Freiburg, Germany; ^4^St. Lukas Klinik, Abteilung für Onkologie und Hämatologie, Ohligs, 42697 Solingen, Germany; ^5^Gemeinschaftspraxis für Strahlentherapie-Bonn-Rhein-Sieg, Bonn, 53177 Bad Godesberg, Germany; ^6^Evangelisches Klinikum Niederrhein gGmbH, 47169 Duisburg, Germany

## Abstract

We analysed trends over time in palliative first-line chemotherapy in patients with locally advanced or metastatic esophagogastric cancer. Special focus was on frequency and quality of HER2-testing and trends in drug use in combination with trastuzumab. Earlier published data about patients treated outside clinical studies showed a relatively low rate of HER2-testing and insufficient test quality. A total of 2,808 patients retrospectively documented in Therapiemonitor^*®*^ from 2006 to 2013 were analysed regarding treatment intensity and trends in used drugs. Data on HER2-testing and therapies were analysed in two cohorts documented in 2010 and 2011 (1) compared to 2012 and 2013 (2). Treatment intensity increased: 49.3% of patients received at least a triplet in 2013 compared to 10.1% in 2006. In cohort 2 HER2 expression was tested in 79.1% of the cases. Still, in 26.9% testing was not done as requested by guidelines. Good performance status, multiple metastases, age ≤ 65 years, the objective “to prevent progression,” good cognitive capabilities, estimated good compliance, and social integration positively influenced the probability of HER2-testing; comorbidities negatively affected it. Usage of the combination of fluoropyrimidines and cisplatin with trastuzumab declined from 67% in cohort 1 to 50% in cohort 2.

## 1. Introduction

Gastric cancer is one of the most common malignancies worldwide, accounting for 950,000 new annual cases worldwide [1] and 15,840 newly diagnosed patients in Germany in 2010 [2].

In Western countries without specialized screening programs late diagnosis is common. Therefore the majority of patients present with locally advanced or metastatic disease and require palliative treatment during the course of their disease.

Several chemotherapy agents have been studied in the first-line therapy of advanced gastric cancer as single agents as well as a part of combination therapies. Among the “older” drugs 5-fluorouracil (5-FU), epirubicin, cisplatin, and mitomycin C are best known substances [3–6].

During past years, oxaliplatin, capecitabine, docetaxel, and irinotecan have been introduced in the treatment of gastric cancer. Oxaliplatin as a combination partner in multiagent therapies has been shown to be as effective as cisplatin [7] with fewer toxicities [8]. The oral fluoropyrimidine capecitabine has been investigated and proved to be a suitable substitute for 5-FU [7]. Taxanes such as paclitaxel and docetaxel have also shown to be effective [9, 10] as single agent and in combinations [11].

Chemotherapy triplets are used more frequently, and even in elderly patients it could be shown that treatment intensification may be reasonable in certain clinical situations [12, 13].

Trastuzumab in combination with chemotherapy proved to be effective in patients whose carcinomas show HER2 overexpression [14] and is thus recommended for these patients [15–17]. Market approval in Germany was granted in 2010.

Standardized immunohistochemical testing (IHC) and in situ hybridisation (ISH) techniques are required to test for HER2 overexpression [18, 19]. HER2 overexpression is defined as either an IHC score of 3+ or an IHC score of 2+ and confirmation of gene amplification using in situ hybridisation (ISH) techniques.

In 2013, first data on the practical use of trastuzumab outside clinical studies in gastric cancer after market approval in Germany collected with Therapiemonitor were reported [20]. Main findings of this analysis were a relatively low testing rate for HER2 of only 49.1% of all documented patients in need for first-line palliative chemotherapy. In addition the recommended test algorithm was applied in only 52.2% of the tested patients.

Here we report data obtained with Therapiemonitor from 2006 to 2013 in patients with advanced esophagogastric cancer with a focus on patients undergoing first-line palliative chemotherapy outside of clinical studies reflecting “real-life” therapy strategies.

We were interested whether changes in drug use and treatment intensity in comparison to the previous reported data occurred. Moreover, we focused on HER2-testing strategies and variables influencing likelihood of testing as well as the quality of the used test algorithms.

## 2. Materials and Methods

### 2.1. Therapiemonitor

Therapiemonitor is a method to collect real world data regarding treatment decisions and strategies in patients with malignant diseases. To achieve this, a retrospective documentation of anonymized clinical and epidemiological data of patients undergoing treatment decisions within a defined timespan is performed in a representative sample of institutions. A detailed description of the methodology has been reported earlier [20, 21]. The selection of centers for documentation follows a two-step procedure. The apportioned and stratified random sample is based on an initial survey among all institutions (about *n* = 800) dealing with the treatment of patients with advanced gastric cancer. According to this survey, the “treated prevalence” is ascertained and a collective of patients is apportioned according to treatment center and distributed regionally according to population density. In a second step, selected centers are asked to document their patients undergoing treatment decisions in the respective time period.

Treatment, demographic- and tumor-related data, former medical and surgical treatment, and socioeconomic data like insurance status available in a patient file are documented by the treating physicians. Response and outcome data such as progression-free or overall survival are not collected. Data are double checked: in a real time automatically and centrally for plausibility and completeness by clinical monitors.

### 2.2. Statistical Methods

All analyses presented herein are explorative. Depending on the size of the predicted numbers and the number of included categories, the Pearson Chi-square, likelihood-quota, or the exact Fisher-test were used.

For all comparisons a *p* value of less than 0.05 was considered statistically significant. Analyses were performed using IBM SPSS Statistics for Windows, Version 19.0 (IBM Corp., Armonk, NY).

## 3. Results

### 3.1. Patient and Tumor Characteristics

Between 2006 and 2013 a total of 2,808 patients with locally advanced, unresectable, or metastatic adenocarcinoma of the stomach or esophagogastric junction and therefore in need for palliative first-line treatment were documented in Therapiemonitor.

Patient and tumor characteristics of all patients are shown in Table 1. Pooled data from the years 2006–2009 and from 2010 have already been published [20, 21]. Gender distribution and median age as well as the main tumor characteristics remained stable across the documented years. A continuous increase of the proportion of patients with Karnofsky performance status (KPS) < 80% receiving first-line chemotherapy is seen with 27.1% in 2006–2009 compared to 33.4% in 2013 (Table 1).

### 3.2. Treatment Intensity, Administered Drugs, and Trends over Time

The use of cisplatin decreased from 51.1 to 31.9% between 2006 and 2013, while oxaliplatin was administered to an increasing number of patients (23.8 to 52.7% reaching a plateau since 2011). Likewise, docetaxel use increased from 20.5 to 35.9% with relatively stable amounts since 2011. The use of capecitabine increased from 12.6% in 2006–2009 up to a maximum of 24.5% in 2011 and showed a slight decrease to 19.3% in 2013. Epirubicin usage peaked in 2010 with 24.7% but decreased to 7.0% in 2013. Paclitaxel, irinotecan, etoposide, and mitomycin continue to play a minor role, if any, in the first-line treatment (Table 2).

Treatment intensity increased in the recent years: the amount of triplet therapies amounted to 49.3% in 2013 (Table 3).

### 3.3. Variables Influencing Treatment Intensity in 2012 and 2013

Data about first-line chemotherapy outside clinical trials documented in 2012 and 2013 was available in 675 patients. The median age of these patients was 65 (range 19–88); sixty-two percent of the patients (*n* = 419) were male. The majority (67.0%) had a good performance status with KPS ≥ 80%. Twenty-one percent (*n* = 141) were treated in university hospitals, fifty-one percent (*n* = 345) in other hospitals, and thirty-six percent (*n* = 246) in oncology practices. As mentioned above these patients characteristics remained stable compared to those previously in Therapiemonitor documented and published patient data.

In these patients first-line chemotherapy consists of chemotherapy alone in 553 patients (81.7%) whereas 121 patients (17.9%) received chemotherapy in combination with trastuzumab and 3 patients (0.4%) received trastuzumab monotherapy. 43.6% (*n* = 295) were treated with single agents or doublet chemotherapy; 56.4% (*n* = 382) received at least triplets. Age <65 years correlated significantly with a more intense treatment (*p* < 0.001). Patients who received at least a triplet therapy were younger (median 63 years, range 21–88) than those who had monotherapy or doublet therapy (68 years, range 19–88). Performance status and presence of concomitant disease were also significant factors influencing treatment intensity: the overall amount of patients with KPS ≥ 80% was 67.0%. The group receiving triplets had a significantly higher amount of good-status patients (74.4%) than the lower intensity group (57.4%, *p* < 0.001) and a significantly lower amount of patients with concomitant disease (51.1% versus 66.8/*p* < 0.001). In addition “achievement of resectability” as objectives of the systemic therapy led to the use of triplets (*p* < 0.001) while other objectives, namely, “prevention of progression,” “improvement of tumour related symptoms and quality of life,” had no impact on the decision to use a more intensive chemotherapy regimen. Also the presence of metastases, assumed compliance and cognitive capabilities or socioeconomic factors like education, social integration or insurance status did not influence treatment decision.

### 3.4. Frequency and Quality of HER2-Testing

A total of 683 patients documented in 2012 and 2013 were evaluable for the analyses of HER2-testing. Only patients outside clinical trials were included. Out of these patients, 79.1% (*n* = 540) were tested for HER2 expression. Of these 89 patients did not undergo an IHC analysis as first step of the test algorithm or the testing method was not reported (*n* = 2). In 142 patients with IHC 0, 1+, or 3+ an ISH analysis was performed whereas in 3 patients with IHC 2+ no ISH analyses were performed adding to a total of *n* = 145 patients in whom the suggested test algorithm was not applied in an appropriate manner (26.9% of tested pts). Regarding the test results in *n* = 449 patients with IHC as first step in the testing algorithm the distribution of IHC scores was as follows: IHC 0 *n* = 196 (43.7%), IHC 1+ *n* = 120 (26.7%), IHC 2+ *n* = 49 (8.9%), and IHC 3+ *n* = 84 (18.7%). Of the *n* = 49 patients with IHC 2+, *n* = 28 had a positive ISH analyses. Taken together, 112 (*n* = 84 with HER3+ and 28 with HER2+/ISH +) out of 540 patients (20.7%) fulfilled the criteria for HER2 positivity and therefore were eligible for trastuzumab treatment. Of these patients 96 were treated with trastuzumab as part of first-line treatment which equals 86% of the eligible patients.

In addition, 20 patients were reported to have HER2 positive tumor and were treated with trastuzumab. However, the test results reported by the treating physicians of these patients in the case report forms did not indicate HER2-positivity. In ten of these patients no IHC analyses as first step of the test algorithm were performed. Another eight patients with IHC 0 and 1+ underwent an ISH analysis indicating HER2 overexpression. In addition in two patients with IHC 2+ no additional ISH analysis was performed.

### 3.5. Factors Influencing the Probability for HER2-Testing

**Figure 1:**
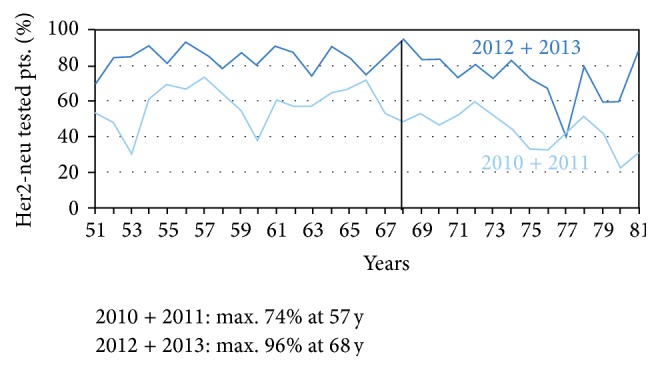
Percentage of patients tested for HER2-expression in relation to age compared in the actual cohort documented in 2010 and 2011 to patients documented in 2012 and 2013. In total in 2010 and 2011 589 of 1123 documented patients (52%) and in 2012 and 2013 540 of 640 (79%) documented patients were tested for HER2-expression.

Tumor specific as well as patient specific and institutional related variables were analysed for the likelihood of HER2-testing. Variables affecting HER2-testing in the actually reported group in 2012 and 2013 compared to previous reported patients in 2010 and 2011 are depicted in Table 4. Significant correlations for a higher likelihood of applying a HER2 test were found for KPS ≥ 80% (*p* < 0.001), the presence of multiple metastases (*p* = 0.007), lower age ≤ 65 years (*p* = 0.048), the objective “to prevent progression” (*p* = 0.006) and patient specific factors assumed by the treating physicians as good cognitive capabilities (*p* < 0.001), good compliance (*p* < 0.001), and complete social integration (*p* = 0.047). Concomitant diseases negatively affected the decision to test for HER2 (*p* = 0.003). The percentage of patients tested for HER2-expression in relation to age compared in the actual cohort documented in 2012 and 2013 to patients documented in 2010 and 2011 is shown in Figure 1. In 2012 and 2013 the maximum of 96% were tested at the age of 68 years compared to a maximum of 74% of patients tested in 2010 and 2011 at the age of 57 years.

Other factors were also analysed: education, insurance status, the treating institution, gender, initial diagnosis with stage IV disease, tumor size, tumor localisation, localisation of metastatic site, or subtype of tumor histology had no impact on the decision to test for HER2 (data not shown).

### 3.6. Chemotherapy Backbone in Combination with Trastuzumab

Different drugs were used as chemotherapy backbone in conjunction with trastuzumab in patients reported in 2010 and 2011 in comparison with the latest cohort in 2012 and 2013. The proportion of patients receiving cisplatin containing regimens decreased from 76% in 2010 and 2011 to 54% in 2012 and 2013 whereas the use of docetaxel containing regimens increased from 13% to 25% as well as the use of oxaliplatin increased from 19% up to 44% in the same time span. The use of capecitabine as a substitute for 5-FU remained stable with 34% and 32%, respectively. In 2010 and 2011 the majority of patients (67%) received trastuzumab in combination with a chemotherapy doublet of cisplatin and 5-FU or capecitabine. In comparison in 2012 and 2013 only 50% of the patients received this combination. The use of 5-FU or capecitabine in combination with oxaliplatin and trastuzumab showed a slight increase from 14% in 2010 and 2011 to 18% in 2012 and 2013. Therefore in total 83% of patients in 2010 and 2011 were treated with trastuzumab and a chemotherapy doublet compared to 68% in 2012 and 2013. Accordingly, the use of trastuzumab in combination with a chemotherapy triplet showed a slight increase from 15% in 2010 and 2011 to 22% in 2012 and 2013. The same applies to the use of trastuzumab in combination with 5-FU or capecitabine or docetaxel monotherapy (4% to 10%). The most frequent used chemotherapy triplet as backbone in combination with trastuzumab was 5-FU/capecitabine in combination with docetaxel and oxaliplatin with 8% in 2010 and 2011 rising up to 19% in 2012 and 2013. The use of cisplatin in combination with docetaxel and 5-FU or capecitabine remained stable with 4% in 2010 and 2011 and 3% in 2012 and 2013.

## 4. Discussion

Here we report on a large cohort of 2,808 patients with metastatic esophagogastric adenocarcinoma who were treated between 2006 and 2013 and documented with a healthcare research tool to collect and analyse data on treatment reality in cancer patients. We focused on trends over time in treatment intensity, used drugs, HER2-neu testing frequency, and quality as well as anti-HER directed treatment patterns. Patient and tumour characteristics of these patients remained stable through the time span from 2006 to 2013. Noteworthily the amount of patients with poor KPS < 80% receiving chemotherapy increases over the years from 27.1% in 2006 to 33.4% in 2013 meaning even frail patients were treated. A possible explanation of this “real-life” trend are the encouraging results in studies as FLOT 65+ performed especially in elderly patients [12, 13].

An ongoing trend to use oxaliplatin instead of cisplatin as well as an increase of the use of docetaxel as part of the 1st-line treatment was observed [20, 21]. Most probably these findings reflect the results of several studies demonstrating the efficacy and feasibility of docetaxel and oxaliplatin containing regimens [8, 11, 22] and as mentioned above in terms of FLOT-65 even in elderly patients [12, 13]. In contrast, epirubicin as part of chemotherapy triplets in the 1st-line setting is less used since 2010. One could speculate this is due to several disadvantages in the application of the most common used epirubicin containing regimens in Germany such as ECF and ECX or EOX like the need for continuous infusion of 5-FU through the whole treatment period or difficulties in swallowing while using an oral application form as capecitabine in patients with esophagogastric cancer. As reported before [20, 21] most of the patients received combination treatment as first-line chemotherapy with half of the patients receiving at least a chemotherapy triplet since 2010 reflecting the actual German and international guidelines for treatment of locally advanced or metastatic esophagogastric cancer [16].

We analysed factors influencing treatment intensity in 675 patients documented in the last cohorts in 2012 and 2013. Age < 65 years, good KPS > 80, lower presence of concomitant disease, and the objective “achievement of resectability” lead to the use of at least a chemotherapy triplet in these patients. This compares adequately with what was observed in previous documented cohorts [20, 21].

Another focus of our present analyses was frequency and quality of HER2-testing in a cohort of 683 patients documented in 2012 and 2013. Almost 80% of the patients were tested for HER2 expression. In 26.9% of the patients the suggested test algorithm was not applied in the appropriate manner. Compared to earlier published data documented in 2010 [20] with only 49.1% of patients tested for HER2-expression and 52.2% of patients with inappropriate test algorithms this represents almost doubling in test rate and “quality” of testing. The most probable reasons for this efforts, especially in test quality, are the widespread availability of testing and commonly adoption of the proposed test algorithm in qualified laboratories as published in 2011 [23, 24]. This may be underlined by the finding that in contrast to earlier published data [20] the treating institution clinics versus office based physicians are no longer a variable affecting the frequency of HER2-testing.

Regarding other variables affecting the likelihood for testing for HER2 expression in the actual cohort (2012 and 2013) the following factors were significant positive predictors in bivariate analyses: higher KPS, good cognitive capabilities, assumed good compliance, the objective “to prevent progression,” the presence of multiple metastases, lower age ≤ 65 years, and complete social integration. Concomitant diseases negatively affected the decision to test for HER2. Again, this compares adequately with what was observed in previous documented cohorts for most of the analysed factors [20]. Noteworthily the factor age ≤ 65 years seems to be a less strong predictor for testing for HER2-expression in our actual cohort in 2012 and 2013 compared to 2010 and 2011 and the maximum of 96% of patients tested at the age of 67 years in 2012 and 2013 compared to 74% of patients at the age of 57 years in 2010 and 2011. A possible explanation of this observation may be found in the analyses of the used chemotherapy backbone in combination with trastuzumab. In the cohort of 2010 and 2011 67% of the patients received trastuzumab according to label in combination with 5-FU and cisplatin whereas in 2012 and 2013 only 50% of the patients were treated with this combination. Accordingly the use of trastuzumab in combination with a 5-FU or docetaxel monotherapy or the use of trastuzumab monotherapy rose from 4% to 10% reflecting the fact the treating physicians consider a less toxic monochemotherapy regimen in combination with trastuzumab or even a trastuzumab monotherapy as an effective treatment regimen even in the elderly and therefore especially these patients showed higher test rates than in previous years. Regarding the ongoing trend for treatment intensification in the good performance status patients the use of a chemotherapy triplet in combination with trastuzumab rose from 15% in 2010 and 2011 to 22% in 2012 and 2013. The most commonly used chemotherapy triplets in combination with trastuzumab in the patients documented in 2012 and 2013 were 5-FU or capecitabine/oxaliplatin/docetaxel in 19% of the patients. Another 18% of the patients received trastuzumab in combination with 5-FU or capecitabine and oxaliplatin confirming previously reported data [20, 21] and the ongoing trend of the use of less toxic oxaliplatin instead of cisplatin as well in combination with trastuzumab. The efficacy and feasibility of the use of oxaliplatin in combination with trastuzumab in the treatment of locally advanced or metastatic gastric cancer were confirmed in a recently published phase II study [25] and a retrospective analyses [26].

## 5. Conclusion

In summary, in the present analysis we found an ongoing trend toward the use of more intensive treatment in first-line chemotherapy in patients with esophagogastric adenocarcinoma. In 2012 and 2013, more than two years after market approval of trastuzumab in the treatment of esophagogastric cancer, HER2-testing is widely used according to the suggested and standardized test algorithms and independent of the treating institutions, reflecting the feasibility of test-based treatment strategies. Half of the patients documented in 2012 and 2013 and treated with trastuzumab outside clinical studies were not treated according to label with cisplatin and 5-FU as chemotherapy combination partners leading to uprising test rates and treatment especially in the elderly.

## Figures and Tables

**Table 1:** Patient and tumor characteristics of patients (*n* = 2.808) with advanced or metastatic esophagogastric adenocarcinoma documented in Therapiemonitor 2006–2013. CT = chemotherapy; KPS = Karnofsky performance status; TT = targeted therapy.

	Total number of patients 2006–2009 *n* (%)	Total number of patients 2010 *n* (%)	Total number of patients II.-III. quarter 2011 *n* (%)	Total number of patients III. quarter 2012 *n* (%)	Total number of patients III. quarter 2013 *n* (%)^§§§^
Total number of patients receiving palliative 1st-line CT/TT (*n*)	1,058	754	314	325	357

Gender^§^					
Male; *n* (%)	674 (63.8)	473 (62.7)	205 (65.3)	200 (61.5)	226 (63.3)
Female; *n* (%)	383 (36.2)	281 (37.3)	109 (34.7)	125 (38.5)	131 (36.7)

Age; median (years)^§§§^	67	67	66	65	65
Range (years)	24–100	24–90	29–96	20–86	19–88
Patients aged < 65 years (%)	44.7	42.3	46.8	46.9	49.7

Patients with KPS ≥ 80% in 1st-line treatment (%)^§§§§§^	72.9	72.6	70.7	67.8	66.6

Patients with initial diagnosis of carcinoma in stage IV (%)	69.8	70.0	65.0	67.7	74.2

Histology					
Signet cell cancer (%)	14.5	24.1	24.2	12.3	26.9
Undifferentiated cancer (G3) (%)	43.5	46.9	43.6	44.0	45.7

Metastatic sites^§§^					
Liver (%)	50.1	62.0	59.5	61.8	61.6
Peritoneum (%)	43.2	45.7	47.3	47.8	55.7
Lung (%)	17.1	24.9	26.7	20.5	20.4
Bone (%)	8.5	10.0	13.8	11.8	8.4

Patients participating in clinical trials on 1st-line chemotherapy (%)	10.1	7.8	4.8	1.5	0.6

Treatment institution^§§,§§§^					
University hospital	16.9	25.2	26.8	17.8	23.9
Other hospitals	59.7	52.4	53.4	60.3	42.4
Oncology practice	28.0	29.6	35.1	33.2	39.3
Unknown	—	—	—	0.6	0.6

Insurance status					
Statutory insurance (%)	91.7	88.8	87.6	88.9	84.3
Private insurance (%)^§§§§^	8.3	11.2	12.4	11.1	15.7

*Note*. ^§^Information on gender is missing in one patient. ^§§^Multiple answers were permitted.

^§§§^Information is missing in one patient 2013. ^§§§§^Other than statutory insurance, ^§§§§§^in 2012 and 2012 the KPS was placed in the last form “Therapiestatus” and changed to KPS in the last therapy decision.

**Table 2:** Anticancer drugs used in the 1st-line treatment of patients advanced or metastatic esophagogastric adenocarcinoma (*n* = 2,803) documented in Therapiemonitor 2006–2013. Indicated is the number of patients receiving the respective drugs in the respective years and/or quarters.

	Total number of patients 2006–2009 *n* (%)	Total number of patients 2010 *n* (%)	Total number of patients II.-III. quarter 2011 *n* (%)	Total number of patients III. quarter 2012 *n* (%)	Total number of patients III. quarter 2013 *n* (%)
Cisplatin	538 (51.1)	370 (49.1)	112 (35.7)	103 (31.7)	114 (31.9)
Oxaliplatin	251 (23.8)	286 (37.9)	161 (51.3)	170 (52.3)	188 (52.7)
Capecitabine	133 (12.6)	177 (23.5)	77 (24.5)	75 (23.1)	69 (19.3)
Docetaxel	216 (20.5)	193 (25.6)	103 (32.8)	112 (34.5)	128 (35.9)
Paclitaxel	6 (0.6)	2 (0.3)	1 (0.3)	—	2 (0.6)
Irinotecan	92 (8.7)	31 (4.1)	12 (3.8)	20 (6.2)	13 (3.6)
Epirubicin	107 (10.2)	186 (24.7)	60 (19.1)	42 (12.9)	25 (7.0)
Mitomycin C	12 (1.1)	5 (0.7)	—	—	—
Etoposide	45 (4.3)	7 (0.9)	3 (1.0)	2 (0.6)	3 (0.8)
Evaluable patients (*n*)	1053	754	314	325	357

**Table 3:** Treatment intensity in the 1st-line treatment of patients with advanced or metastatic esophagogastric adenocarcinoma (*n* = 2,803) documented in Therapiemonitor 2006–2013. Indicated is the percentage of patients receiving the respective treatment in the indicated years and/or quarters.

	2008 (%)	2009 (%)	2010 (%)	2011 (%)	2012 (%)	2013 (%)^§^
Monochemotherapy	11.8	6.4	6.1	7.3	7.7	10.1
Chemotherapy doublet	57.7	46.6	33.7	29.0	32.9	35.9
Chemotherapy triplet	30.5	47.0	58.4	60.2	54.2	49.3
Chemotherapy > triplet	—	—	1.9	3.5	5.2	6.7

*Note*. Folinic acid is not considered an active drug and is consequently not included in this analysis.

^§^In 2013 folinic acid was asked as a separate drug, but see *Note* above.

**Table 4:** Chi-square test for variables with potential predictive value regarding the likelihood of HER2-testing. Included in the analysis are only patients not participating in clinical trials (2010 + 2011: *n* = 1123; 2012 + 2013: *n* = 684). AEG = adenocarcinoma of the esophagogastric junction.

Variable	2010 + 2011 *p* value	2012 + 2013 *p* value
Karnofsky performance status ≤80 versus >80	<0.001	0.391
Age ≤ 65	<0.001	0.039
Number of metastases: none/singular versus multiple	<0.001	0.008
Treated concomitant disease: yes versus none	0.025	0.003
Objective of treatment: “resectability of the primary tumor”: yes versus no	0.023	0.275
Objective of treatment: “prevention of progression”: yes versus no	0.003	0.006

*Note*. The included patients differ according to the valid answers.
